# Predictors of tuberculosis and human immunodeficiency virus co-infection: a case-control study

**DOI:** 10.4178/epih/e2014024

**Published:** 2014-10-30

**Authors:** Leila Molaeipoor, Jalal Poorolajal, Minoo Mohraz, Nader Esmailnasab

**Affiliations:** 1 Department of Epidemiology and Biostatistics, School of Public Health, Hamadan University of Medical Sciences, Hamadan, Iran; 2 Research Center for Health Sciences and Department of Epidemiology and Biostatistics, School of Public Health, Hamadan University of Medical Sciences, Hamadan, Iran; 3 Iranian Research Center for HIV/AIDS, Iranian Institute for Reduction of High-Risk Behaviors, Tehran University of Medical Science, Tehran, Iran; 4 Kurdistan Research Center for Social Determinants of Health, School of Medicine, Kurdistan University of Medical Sciences, Sanandaj, Iran

**Keywords:** Human immunodeficiency virus, Acquired immunodeficiency syndrome, Tuberculosis, Risk factors, Case-control studies, Iran

## Abstract

**OBJECTIVES:**

The human immunodeficiency virus (HIV) and *Mycobacterium tuberculosis* co-infection is a major global challenge. It is not clear why some HIV-positive people are co-infected with tuberculosis (TB) while others are not. This study addressed that question.

**METHODS:**

This case-control study was conducted in Tehran, Iran in June 2004, enrolling 2,388 HIV-positive people. Cases were selected from those who were co-infected with TB and controls from those without TB. Multiple logistic regression analysis was performed to assess the association between *M. tuberculosis*/HIV co-infection and several predictors. Odds ratios (ORs) and their 95% confidence intervals (CIs) were calculated.

**RESULTS:**

In this study, 241 cases were compared with 2,147 controls. Sex, age, marital status, educational level, imprisonment, smoking, narcotic use, route of HIV transmission, previous TB infection, isoniazid preventive therapy (IPT), antiretroviral therapy (ART), and low CD4 count (<350 cells/mm^3^) were independently associated with *M. tuberculosis*/HIV co-infection (p<0.001). However, after adjusting for all other variables in the model, only the association between *M. tuberculosis*/HIV co-infection and the following predictors remained statistically significant: imprisonment (odds ratio [OR], 3.82; 95% confidence interval [CI], 2.11-6.90), previous TB infection (OR, 5.54; 95% CI, 1.99-15.39), IPT (OR, 0.13; 95% CI, 0.06-0.31), ART (OR, 1.81; 95% CI, 1.26-2.61), and CD4 count <350 cells/mm^3^ (OR, 2.34; 95% CI, 1.36-4.02).

**CONCLUSIONS:**

Several predictors are associated with *M. tuberculosis*/HIV co-infection, but only a few indicators were significantly associated with *M. tuberculosis*/HIV co-infection. It is estimated that a number of predictors of *M. tuberculosis*/HIV co-infection remain unknown and require further investigation.

## INTRODUCTION

To halt and reverse the tuberculosis (TB) epidemic by 2015 is the sixth Millennium Development Goals [1]. The human immunodeficiency virus (HIV)/acquired immunodeficiency syndrome (AIDS) and *Mycobacterium tuberculosis* co-infection is one of the major global challenges in the control of TB [2]. The risk of developing TB is 20-37 times greater in HIV-positive people than HIV-negative people [3]. TB is the leading preventable cause of death among HIV-infected people [2] and is responsible for more than 25% of deaths in people living with HIV [3]. TB increases the risk of HIV-related mortality by 4.5fold [4]. In 2012, there were an estimated 8.6 million new cases of TB and 1.3 million died from TB. Over 95% of TB cases and deaths occur in low- and middle-income countries [5].

There are several conditions associated with increased risk of *M. tuberculosis*/HIV co-infection. Men living with HIV are more likely to be infected with TB than women [6-8]. The TB prevalence shows a bimodal distribution at the extremes of age [9]. The active TB is more common in immunosuppressed people [5]. People who live in close contact with people who have active TB are more likely to be infected with TB [10]. In HIV-positive people, the probability of TB increases as the CD4 cell count decreases [7,8,10,11]. Tobacco use is substantially associated with increased risk of disease and mortality caused by TB [5]. Anemia and iron deficiency disorders are associated with an increased risk of TB [12,13]. Low body mass index can increase the probability of the TB infection [14,15].

Several studies have been conducted worldwide to assess the predictors of *M. tuberculosis*/HIV co-infection [9,11-15]. However, the prognostic factors behind the breakdown in immune defenses associated with TB infection in people living with HIV are not fully understood. It is unclear why some HIV-positive people are co-infected with TB while others are not. Until reliable information on predictors of TB is collected, it will remain difficult to design effective preventive intervention strategies to control TB in HIV-infected people. This case-control study was conducted in a large population in Iran to determine the main prognostic factors associated with HIV and *M. tuberculosis* co-infection.

## MATERIALS AND METHODS

This case-control study was conducted in the megacity of Tehran, Iran in June 2004. The Research Council of Hamadan University of Medical Sciences approved the study. The study population was HIV-positive people with or without TB who had a medical record in one of two Behavioral Diseases Counseling Centers, the Imam Khomeini and Zamzam Centers. These centers provide several health services, such as education, counseling, self-care, prevention, treatment, and harm reduction, to people who are at high risk of HIV and sexually transmitted diseases [16].

The cases were selected from HIV-positive people co-infected with TB aged equal to or greater than 18 years irrespective of sex and date of diagnosis, whether or not they had developed AIDS. The additional data on TB infection were extracted from the Centers for Disease Control and Prevention. The controls were selected from HIV-positive people without TB. The controls were selected from a control group from the same database from which the cases were selected in order to make the study base of the two groups the same.

The data collection was done using a checklist of items which was developed according to the information documented in the medical records including demographic information (age, sex, marital status, and educational level), behavioral information (narcotic/alcohol abuse, smoking, and imprisonment), and additional information such as co-infection with hepatitis C virus (HCV), history of previous TB infection, anti-TB prophylaxis, antiretroviral therapy (ART), and CD4 cell count per cubic millimeter. The people with school education (less than or equal to 12 years education) was considered as less educated and those with university education (more than 12 years education) were considered as highly educated.

An HIV-positive subject was defined as an individual with HIV infection irrespective of clinical stage confirmed by laboratory criteria according to country definitions and requirements [17]. In the Islamic Republic of Iran, a case of HIV is defined as a positive result for HIV antibody by two sequential enzyme-linked immunosorbent assay tests confirmed by a western blot test [18]. A case of AIDS was defined as a presumptive or definitive diagnosis of stage 3 or 4 conditions and/or a CD4 count less than 350 cells/mm^3^ of blood in an HIV-infected subject [17].

Active TB was defined as a disease that is caused by *M. tuberculosis* in any part of the body and that is in an active state. In the Islamic Republic of Iran, a patient with two positive sputum smears or one positive sputum smear plus a positive sputum culture or a chest X-ray demonstrating pulmonary TB is considered to have sputum-positive pulmonary TB. A patient with two negative sputum smears but with a positive sputum culture or with signs and symptoms of pulmonary TB not responding to a two-week broad-spectrum antibiotic treatment is considered to have sputum-negative pulmonary TB. Extra-pulmonary TB is diagnosed with bacteriological evidence (positive culture) and pathological findings (caseous necrosis) [19].

Adults and adolescents living with HIV who are unlikely to have active TB, based on a clinical algorithm screening, should receive isoniazid preventive therapy (IPT) for at least 6 months (10 mg/kg/d) as part of a comprehensive package of HIV prevention and care services [2]. In the Islamic Republic of Iran, after excluding active TB, HIV-positive patients with the following criteria will receive IPT: (a) TB skin test (purified protein derivative skin test) equal to or greater than 5 mm; (b) close contact with an infectious TB case; and (c) chest X-ray findings suggestive of fibrotic lesions consistent with TB [16].

Logistic regression analysis was performed to determine the factors predictive of *M. tuberculosis*/HIV co-infection. To control the effect of potential confounding factors, a backward stepwise adjusted analysis was performed to fit the data well and to exclude unnecessary variables from the model. For this purpose, we started with the full model and then excluded variables one at a time, while using the likelihood ratio test to check whether the reduced model or the full model fitted the data significantly well. Crude and adjusted odds ratios (ORs) were reported to address the effect of predictors associated with *M. tuberculosis*/HIV co-infection. All statistical analyses were performed at a significance level of 0.05 using Stata version 11 (StataCorp, College Station, TX, USA).

## RESULTS

We identified 2,519 patients; 25 were ineligible, 21 had medical records in both centers, and 85 were aged below 18 years. The analysis was based on data from the remaining 2,388 people (241 cases and 2,147 controls), of which 1,889 were men and 499 were women. The mean standard deviation age of the patients was 34.99 (8.84) years and ranged from 18 to 74 years.

Results from the bivariate logistic regression analysis of predictors associated with *M. tuberculosis*/HIV co-infection are given in Table 1. There was a highly significant association between male sex and *M. tuberculosis*/HIV co-infection. The risk of TB increased significantly with age. Compared to married people, the single, divorced, or widowed people were at higher risk of infection with TB. The risk of co-infection with TB was higher in highly educated individuals than in those with less education. Imprisonment, smoking, and narcotic use were associated with increased the risk of *M. tuberculosis*/HIV co-infection significantly. Compared to the sexual route of infection, intravenous drug use and multiple routes of HIV transmission were associated with an increased risk of TB. Heterosexuality increased the risk of TB more than other modes of sexual transmission. History of previous TB infection was the strongest risk factor for *M. tuberculosis*/HIV co-infection. The HIV-positive individuals who were co-infected with HCV had a greater risk of co-infection with TB as well. Preventive treatment with isoniazid decreased the risk of TB to less than one-fifth in HIV-positive people. The CD4 cell count less than 350 cells/mm^3^ was associated with an increased risk of TB so that the lower the CD4 cell count, the higher the risk of *M. tuberculosis*/HIV co-infection.

Results from the multivariate logistic regression analysis of predictors associated with *M. tuberculosis*/HIV co-infection are given in Table 2. The pseudo R^2^ statistic, which assesses the predictive strength of the logistic regression, was 0.1444. In order to control the effect of potential confounding and to fit the data well, a backward stepwise adjusted analysis was performed, and the unnecessary variables were excluded from the model. Eight variables remained in the final model, of which only five predictive factors were strongly associated with *M. tuberculosis*/HIV co-infection including imprisonment, previous TB infection, IPT, ART, and reduced CD4 cell count.

## DISCUSSION

Reducing the TB pandemic by 2015 is the sixth Millennium Development Goals [1]. To better understand the risk factors of TB infection in people who live with HIV, the association between several predictors and *M. tuberculosis*/HIV co-infection was investigated. We found that sex, age, marital status, educational level, smoking, narcotic use, and route of HIV transmission were independently associated with increased risk of TB infection in people living with HIV. However, the multivariate analysis revealed no association between these variables and *M. tuberculosis*/HIV co-infection. On the other hand, there was a strong association between *M. tuberculosis*/HIV co-infection and imprisonment, previous TB infection, IPT, ART, and CD4 cell count less than 350 cells/mm^3^.

Current studies have reported that the risk of *M. tuberculosis*/HIV co-infection increases with male sex [6-8], age [9], and tobacco use [5], as was the case in our study. However, multivariate analysis revealed that the associations between *M. tuberculosis*/HIV co-infection and male sex, age, and smoking were confounded by other variables. According to our results, age and smoking do not increase the risk of TB infection in HIV-positive people. On the other hand, current studies have shown that the risk *M. tuberculosis*/HIV co-infection is higher among people who are immunosuppressed [5] or live in close contact with people who have active TB [10] or have a CD4 cell count below 200 cells/mm^3^ [10,11]. Our results were consistent with these findings. Multivariate analysis indicated that previous TB infection and CD4 cell count less than 350 cells/mm^3^ substantially increased the risk of TB in people living with HIV.

We noted that previous imprisonment was related to an increased risk of *M. tuberculosis*/HIV co-infection. Inmates are at a high risk of TB infections because they generally receive minimal preventive care and access to treatment facilities [20]. In addition, inadequate health services may increase the risk of unmanageable HIV and TB epidemics in prisons [21,22].

According to our results, IPT was the most efficient intervention to prevent TB in people who live with HIV and could reduce the risk of TB by 87%. This finding is consistent with current evidence. Current studies have shown that IPT can significantly reduce the risk of TB in people living with HIV in various settings, especially in TB-endemic settings [23,24].

We reported an inverse association between the prevalence of *M. tuberculosis*/HIV co-infection and CD4 cell count, such that the risk of co-infection increased as the CD4 cell count decreased. This finding is consistent with the current evidence [10, 11]. On the other hand, we found that HIV-positive people who received ART were at a higher risk of *M. tuberculosis*/HIV co-infection. The reason is straightforward. ART was initiated for the HIV patients when they developed stage 3 or 4 characteristics or their CD4 cell count decreased to below 350 cells/μL. Both of these conditions can increase the risk of *M. tuberculosis*/HIV co-infection [7,8,10,11].

According to our results, high risk behaviors were more common in cases than in controls. These included imprisonment (80.08% vs. 60.92%), narcotic use (66.80% vs. 51.47%), alcohol use (6.36% vs. 4.15%), smoking (69.05% vs. 55.13%), and previous history of TB infection (4.15% vs. 0.75%). These high risk behaviors might predispose HIV patients to TB infection.

This study had a few limitations. First, this study was performed on data recorded at the Behavioral Diseases Counseling Centers. The quality and accuracy of the associations reported in this study primarily depend on the quality of the recorded data, but we were unable to verify the accuracy of the data. This raises the possibility of information bias. Second, despite several predictors assessed in this study, the value of the pseudo R2 statistic that indicates the predictive strength of the logistic regression was low. This demonstrates that there are still many unknown factors that affect the incidence of TB in people with HIV. Despite its limitations, this study was conducted on a large dataset in the megacity of Tehran, which comprises about 20% of the Iranian population. Therefore, the results of this study may be generalized to a majority of the Iranian HIV-infected population. Furthermore, the effects of several predictive factors on *M. tuberculosis*/HIV co-infection were evident in a middle income country. Such information may be useful for establishing preventive measures.

According to the results of this study, despite the fact that several modifiable and non-modifiable predictors were independently associated with *M. tuberculosis*/HIV co-infection, it was evident that only a few indicators such as imprisonment, previous TB infection, IPT, ART, and low CD4 cell count (<350 cells/ mm^3^) had a significant effect on *M. tuberculosis*/HIV co-infection. In addition, there are still several unknown predictors that affect the association between HIV and TB and require further investigation.

## Figures and Tables

**Table 1.** Results from the bivariate logistic regression analysis of binary and continuous predictors of *M. tuberculosis*/HIV co-infection using unadjusted odds ratios (ORs)

Variables	TB cases (HIV+/TB+)^1^ (n=241)	Non-TB cases (HIV+/TB-)^2^ (n=2,147)	Unadjusted OR (95% CI)	p-value
Gender				
Female	19 (7.88)	480 (22.36)	1.00	
Male	222 (92.12)	1,667 (77.64)	3.36 (2.08-5.43)	0.001
Age (yr)				
18-29	47 (19.50)	636 (29.75)	1.00	
30-39	111 (46.06)	956 (44.71)	1.57 (1.10-2.24)	0.01
≥40	83 (34.44)	546 (25.54)	2.06 (1.41-3.00)	0.001
Marital status				
Married	76 (32.76)	832 (40.88)	1.00	
Single	101 (43.53)	782 (38.43)	1.41 (1.03-1.93)	0.03
Divorced	44 (18.97)	315 (15.48)	1.53 (1.03-2.27)	0.03
Widowed	11 (4.74)	106 (5.21)	1.14 (0.58-2.21)	0.71
Educational level				
High (>12 yr)	8 (3.72)	146 (7.97)	1.00	
Low (≤12 yr)	207 (96.28)	1,685 (92.03)	2.24 (1.08-4.63)	0.03
Imprisonment				
No	48 (19.92)	839 (39.08)	1.00	
Yes	193 (80.08)	1,308 (60.92)	2.58 (1.86-3.58)	0.001
Smoker				
No	65 (30.95)	853 (44.87)	1.00	
Yes	145 (69.05)	1,048 (55.13)	1.82 (1.34-2.47)	0.001
Narcotic use				
No	80 (33.20)	1,042 (48.53)	1.00	
Yes	161 (66.80)	1,105 (51.47)	1.90 (1.43-2.51)	0.001
Alcohol use				
No	221 (93.64)	2,031 (95.85)	1.00	
Yes	15 (6.36)	88 (4.15)	1.57 (0.89-2.76)	0.12
Route of HIV transmission				
Sexual	21 (8.71)	494 (23.01)	1.00	
Intravenous drug users	116 (48.13)	751 (34.98)	3.63 (2.25-3.86)	0.001
Transfusion	5 (2.07)	66 (3.07)	1.78 (0.65-4.89)	0.26
Tattoos	2 (0.83)	26 (1.21)	1.81 (0.40-8.13)	0.44
Surgery or dentistry	2 (0.83)	20 (0.93)	2.35 (0.52-10.73)	0.27
Multiple exposure	87 (36.10)	680 (31.67)	3.01 (1.84-4.91)	0.001
Unknown	8 (3.32)	110 (5.12)	1.71 (0.74-3.96)	0.21
Sexual route of transmission				
Spouse	15 (16.85)	358 (34.52)	1.00	
Heterosexual	68 (76.40)	597 (57.57)	2.72 (1.53-4.83)	0.001
Homosexual	1 (1.12)	26 (2.51)	0.92 (0.12-7.22)	0.93
Bisexual	5 ( 5.62)	56 (5.40)	2.13 (0.75-6.09)	0.16
Previous TB infection				
No	231 (95.85)	2,131 (99.25)	1.00	
Yes	10 (4.15)	16 (0.75)	5.77 (2.59-12.85)	0.001
Hepatitis C virus				
No	111 (46.06)	1,253 (58.36)	1.00	
Yes	130 (53.94)	894 (41.64)	1.64 (1.26-2.15)	0.001
Isoniazid preventive therapy				
No	234 (97.10)	1,811 (84.35)	1.00	
Yes	7 (2.90)	336 (15.65)	0.16 (0.08-0.35)	0.001
Antiretroviral therapy				
No	1,356 (63.16)	136 (56.43)	1.00	
Yes	791 (36.84)	105 (43.57)	1.32 (1.01-1.73)	0.04
CD4 cell count (cells/mm^3^)				
≥350	33 (16.92)	656 (38.54)	1.00	
201-249	34 (17.44)	380 (22.33)	1.78 (2.66-6.62)	0.02
50-200	74 (37.95)	410 (24.09)	3.59 (2.34-5.51)	0.001
<50	54 (27.69)	256 (15.04)	4.19 (2.66-6.61)	0.001
Trend test for strata of CD4 count	241 (100.00)	2,147 (100.00)	1.64 (1.43-1.88)	0.001

HIV, human immunodeficiency virus;TB, tuberculosis; CI, confidence interval.

1 HIV+/TB+, human immunodeficiency virus positive patients with *Mycobacterium tuberculosis* co-infection;

2 HIV+/TB-, human immunodeficiency virus positive patients without *Mycobacterium tuberculosis* co-infection.

**Table 2.** Results from the multivariate logistic regression analysis of binary and continuous predictors of *M. tuberculosis/HIV* co-infection using odds ratios (ORs) adjusted for all other variables in the model (pseudo R^2^=0.144)

Variables	TB cases (HIV+/TB+)^1^ (n=241)	Non-TB cases (HIV+/TB-)^2^ (n=2,147)	Adjusted OR (95% CI)	p-value
Marital status				
Married	76 (32.76)	832 (40.88)	1.00	
Single	101 (43.53)	782 (38.43)	0.95 (0.63-1.45)	0.82
Divorced	44 (18.97)	315 (15.48)	1.09 (0.66-1.81)	0.74
Widow	11 (4.74)	106 (5.21)	1.57 (0.72-3.39)	0.25
Educational level				
High (>12 yr)	8 (3.72)	146 (7.97)	1.00	
Low (≤12 yr)	207 (96.28)	1,685 (92.03)	0.93 (0.43-2.05)	0.86
Imprisonment				
No	48 (19.92)	839 (39.08)	1.00	
Yes	193 (80.08)	1,308 (60.92)	3.82 (2.11-6.90)	0.001
Smoker				
No	65 (30.95)	853 (44.87)	1.00	
Yes	145 (69.05)	1,048 (55.13)	1.27 (0.77- 2.10)	0.36
Previous TB infection				
No	231 (95.85)	2,131 (99.25)	1.00	
Yes	10 (4.15)	16 (0.75)	5.54 (1.99- 15.39)	0.001
Isoniazid preventive therapy				
No	234 (97.10)	1,811 (84.35)	1.00	
Yes	7 (2.90)	336 (15.65)	0.13 (0.06-0.31)	0.001
Antiretroviral therapy				
No	1,356 (63.16)	136 (56.43)	1.00	
Yes	791 (36.84)	105 (43.57)	1.81 (1.26-2.61)	0.001
CD4 cell count (cells/mm^3^)				
≥350	33 (16.92)	656 (38.54)	1.00	
201-249	34 (17.44)	380 (22.33)	2.15 (1.25-3.72)	0.006
50-200	74 (37.95)	410 (24.09)	3.31 (2.01-5.45)	0.001
<50	54 (27.69)	256 (15.04)	4.57 (2.69-7.76)	0.001
Trend test for strata of CD4 count	241 (100.00)	2,147 (100.00)	1.70 (1.45-1.99)	0.001

HIV, human immunodeficiency virus; TB, tuberculosis; CI, confidence interval.

1 HIV+/TB+, human immunodeficiency virus positive patients with *Mycobacterium tuberculosis* co-infection;

2 HIV+/TB-, human immunodeficiency virus positive patients without *Mycobacterium tuberculosis* co-infection.
